# Supplementary material for: A Walk in the Park? Examining the Impact of App-Based Weather Warnings on Affective Reactions and the Search for Information in a Virtual City
Source: Int J Environ Res Public Health. 2021 Aug 6;18(16):8353. doi: 10.3390/ijerph18168353 (PMC8392799; doi:10.3390/ijerph18168353)

Supplementary file S1 - Vignettes

This supplementary file S1 presents the vignettes used in the study. For each time point, two vignettes were used in accordance with one video clip, which is presented in supplementary file S2. The vignettes are listed in order of the experimental procedure depicted in figure 2 in the main text, beginning with a baseline (t1) tour of a virtual city, followed by a warning/no warning (t2), and then a thunderstorm/no thunderstorm in a virtual park (t3):

- Baseline: introduction to the virtual city tour and the app (t1; groups 1-8)
- Warning: virtual city tour interrupted by a weather warning (t2; groups 3,4,7,8)
- No warning: virtual city tour not interrupted by a weather warning (t2; groups 1,2,5,6)
- Thunderstorm: virtual park and thunderstorm (t3; groups 2,4,6,8)
- No thunderstorm: virtual park without thunderstorm (t3; groups 1,3,5,7)

Baseline: introduction to the virtual city tour and the app (t1)

Sie laufen über einen kleinen Marktplatz auf eine Löwenstatue zu.

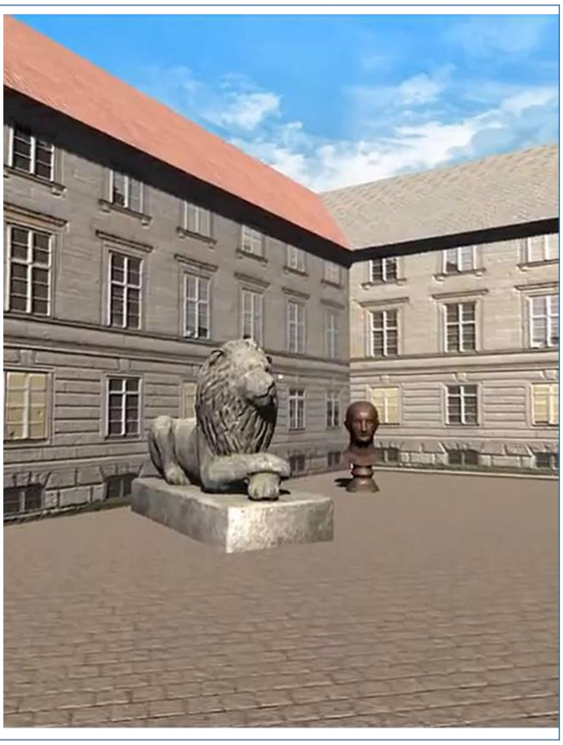

Sie erhalten eine Nachricht auf Ihrem Handy von der App.

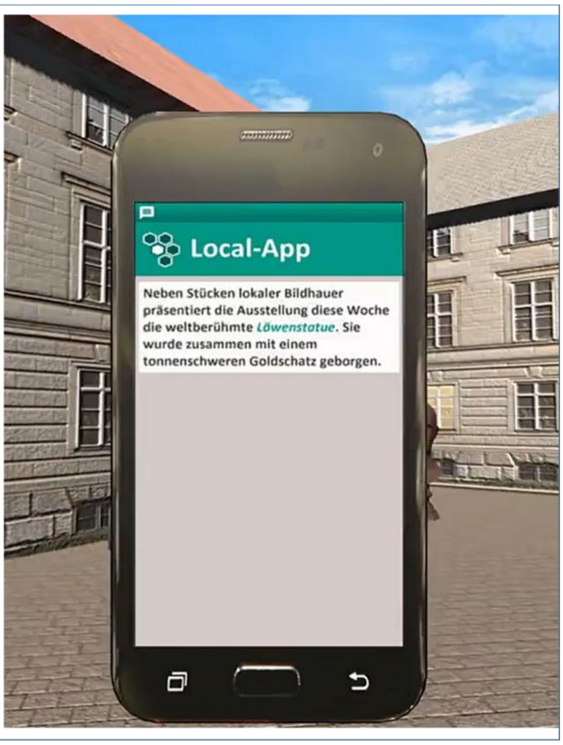

Warning: virtual city tour interrupted by a weather warning (t2)

Sie laufen auf einen großen Platz mit einem Springbrunnen zu.

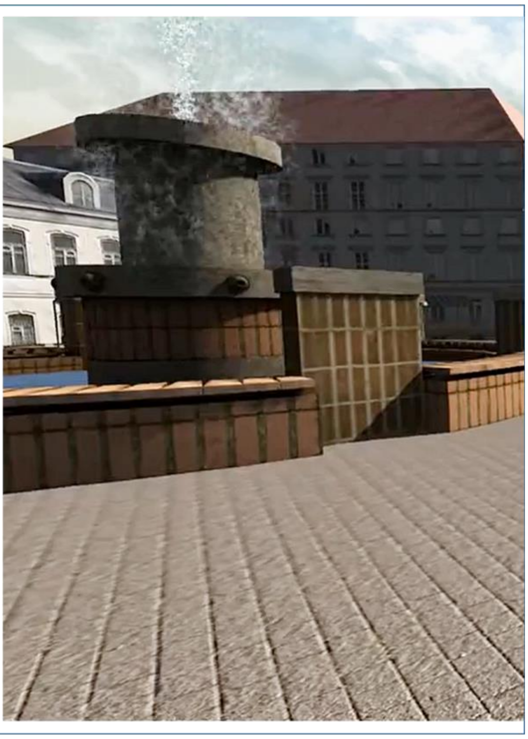

Sie erhalten eine Nachricht auf Ihrem Handy von der App.

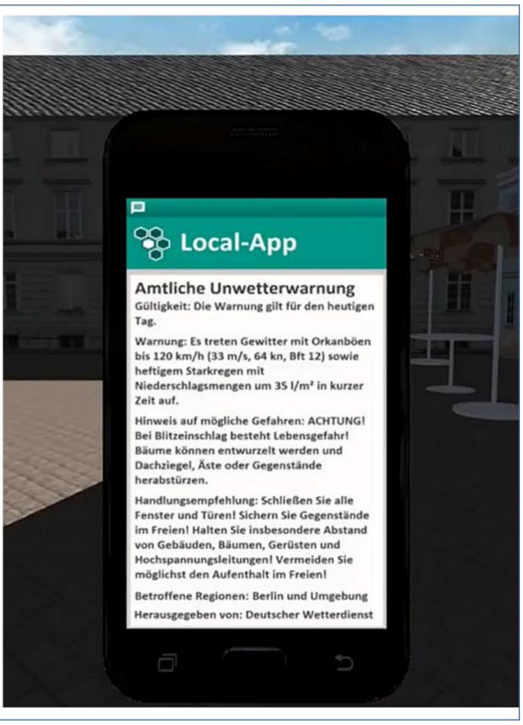

No warning: virtual city tour not interrupted by a weather warning (t2)

Sie laufen auf einen großen Platz mit einem Springbrunnen zu.

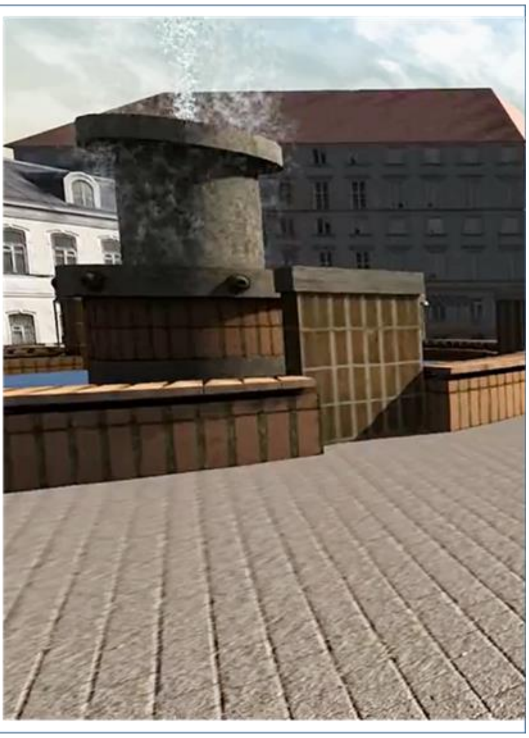

Sie erhalten eine Nachricht auf Ihrem Handy von der App.

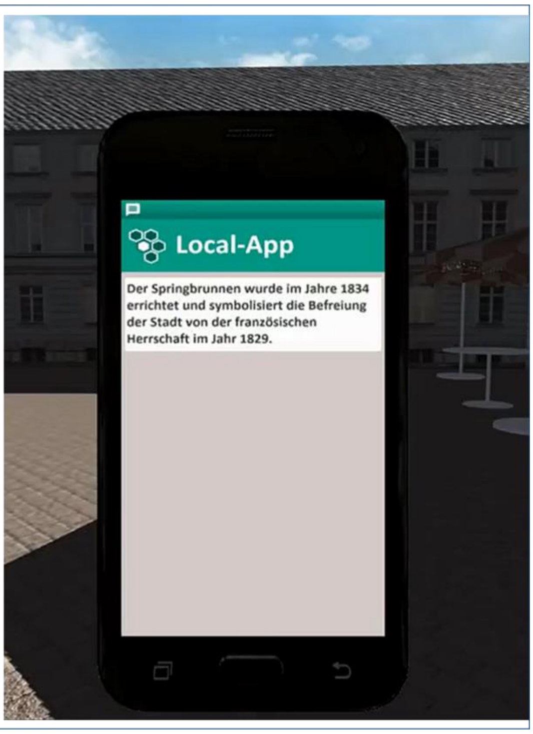

Thunderstorm: virtual park and thunderstorm (t3)

Sie erreichen den Stadtpark und laufen auf einen Blumengarten mit Pavillon zu.

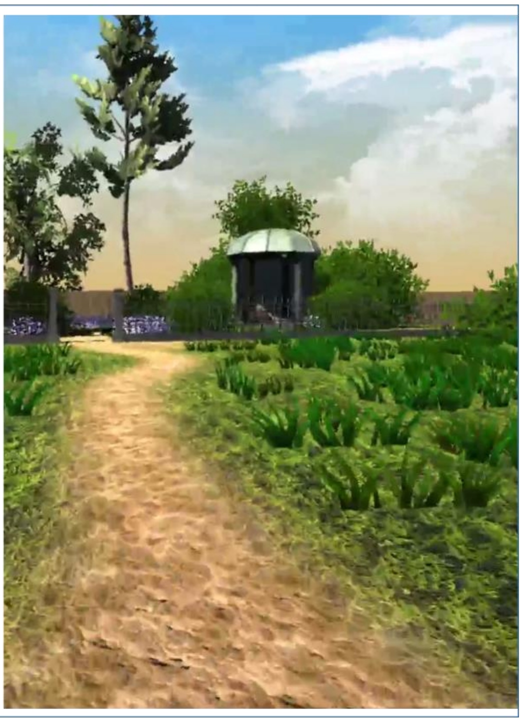

Es beginnt ein Starkregen. Windböen treten und dunkle Wolken ziehen auf. Um Sie herum nehmen Sie Blitze und Donner war.

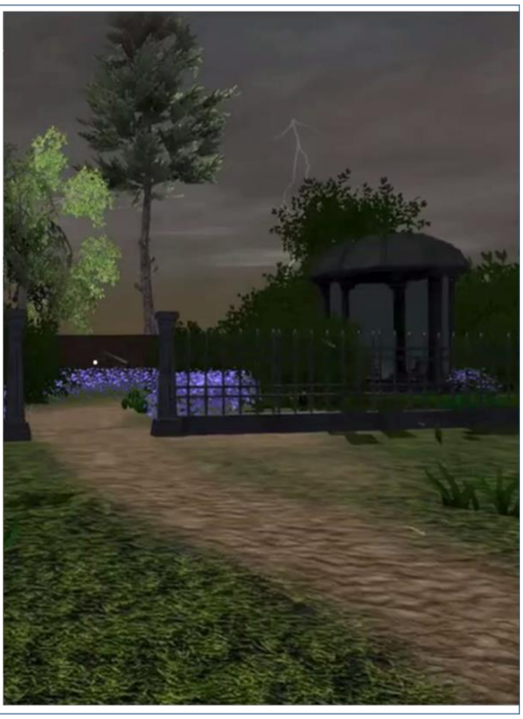

No thunderstorm: virtual park without thunderstorm (t3)

Sie erreichen den Stadtpark und laufen auf einen Blumengarten mit Pavillon zu.

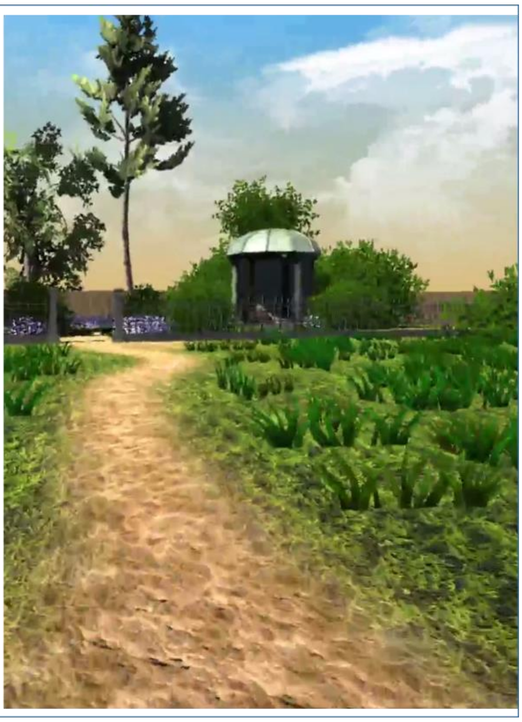

Sie erreichen den Stadtpark und laufen auf einen Blumengarten mit Pavillon zu.

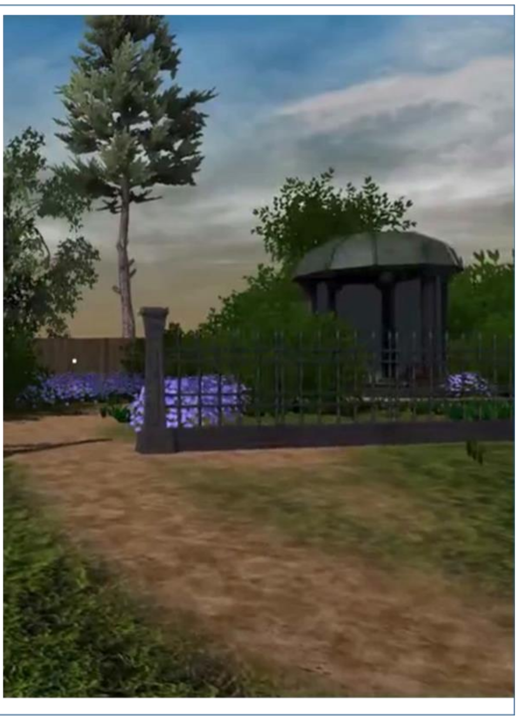

Supplement: Supplementary file 1 [file ijerph-18-08353-s001.zip › Supplementary file S1.pdf]
