# Supplementary material for: A Walk in the Park? Examining the Impact of App-Based Weather Warnings on Affective Reactions and the Search for Information in a Virtual City
Source: Int J Environ Res Public Health. 2021 Aug 6;18(16):8353. doi: 10.3390/ijerph18168353 (PMC8392799; doi:10.3390/ijerph18168353)
Supplement: Supplementary file 1 [file ijerph-18-08353-s001.zip › Supplementary file S2.pdf]

**Supplementary file S2 - Videos**

This supplementary file S2 presents the video clips used in the study. For each time point, a video clip was presented. The videos are listed in order of the experimental procedure depicted in figure 2 in the main text, beginning with a baseline (t1) tour of a virtual city, followed by a warning/no warning (t2), and then a thunderstorm/no thunderstorm in a virtual park (t3):

- Baseline: introduction to the virtual city tour and the app (t1; groups 1-8)
- Warning: virtual city tour interrupted by a weather warning (t2; groups 3,4,7,8)
- No warning: virtual city tour not interrupted by a weather warning (t2; groups 1,2,5,6)
- Thunderstorm: virtual park and thunderstorm (t3; groups 2,4,6,8)
- No thunderstorm: virtual park without thunderstorm (t3; groups 1,3,5,7)
